# High Estimated Glomerular Filtration Rate and Risk of Cancer Mortality in a Japanese Cohort Study: The Ibaraki Prefectural Health Study

**DOI:** 10.31662/jmaj.2022-0120

**Published:** 2022-09-26

**Authors:** Kei Nagai, Toshimi Sairenchi, Kunihiro Yamagata, Kazumasa Yamagishi, Hiroyasu Iso, Fujiko Irie

**Affiliations:** 1 Department of Nephrology, Faculty of Medicine, University of Tsukuba, Tsukuba, Japan; 2 Department of Nephrology, Hitachi General Hospital, Hitachi, Japan; 3 Medical Science of Nursing, Dokkyo Medical University School of Nursing, Shimotsuga, Japan; 4 Department of Public Health, Faculty of Medicine, and Health Services Research and Development Center, University of Tsukuba, Tsukuba, Japan; 5 Institute of Global Health Policy Research (iGHP), National Center for Global Health and Medicine, Tokyo, Japan; 6 Tsuchiura Public Health Center of Ibaraki Prefectural Government, Tsuchiura, Japan

**Keywords:** cancer, renal function, mortality

Cancer is a major cause of mortality and places an enormous burden on both more and less economically developed societies. The occurrence of cancer is increasing because of the growth and aging of populations ^(1)^. In addition, cancer is now the leading cause of mortality in the Japanese general population ^(2)^. While cancer risk in patients with renal impairment remains controversial, large cohort studies have found an association between low estimated glomerular filtration rate (eGFR) and elevated risks of cancer incidence and mortality ^(3), (4), (5), (6), (7)^. Similarly, both higher and lower eGFR were recently considered risk factors for cancer, with a general J-shaped association between eGFR and cancer incidence ^(4), (6)^. The lowest risk of cancer was observed at eGFRs of approximately 45-89 mL/min/1.73 m^2^ and 45-59 mL/min/1.73 m^2^ in a study from the USA ^(6)^ and Korea study after multivariable adjustment, respectively ^(5)^. High eGFR also represented a strong independent predictor of cardiovascular events in a large, multiethnic study, including a Japanese subpopulation ^(8)^. Conversely, current evidence does not support direct associations between high eGFR and the development or progression of cancer. However, risk factors, e.g., diabetes ^(9)^, obesity ^(10)^, and smoking ^(11)^ are associated with high eGFR and are also known risk factors for cancer mortality ^(12)^. The present study, therefore, aims to investigate the association between elevated eGFR and cancer mortality in a large Japanese cohort with long-term follow-up.

The current study examined 89,550 residents (30,366 men [mean age, 60.2 years old]; 59,184 women [mean age, 57.8 years]) in Ibaraki Prefecture who participated in annual community-based health checkups beginning in 1993 at 40-80 years old and who were followed-up through December 2018. The maximum participation duration was 25.7 years. Details regarding the methods applied for mortality surveillance have been previously reported ^(13)^. Briefly, underlying causes of mortality were coded following the International Classification of Diseases, 9th (ICD-9; from 1993 to 1994) or 10th (ICD-10; from 1995) revision. As background, mean values and the prevalence of potential confounding factors were evaluated among patients in four eGFR categories (chronic kidney disease [CKD] stage: G1, ≥90 mL/min/1.73 m^2^; G2, 60-89 mL/min/1.73 m^2^; G3a, 45-59 mL/min/1.73 m^2^; and G3b or worse, <45 mL/min/1.73 m^2^; Table 1). While the rate of current smokers was comparatively high among people with eGFR of ≥90 mL/min/1.73 m^2^, most risk factors for mortality (e.g., body mass index, blood pressure, and lipid status) tended to worsen as renal dysfunction progressed.

**Table 1.** Characteristics of the Study Cohort at Baseline.

eGFR category (mL/min/1.73 m^2^)			≥90	60-89	45-59	<45
	Men					
Study size		(Persons)	3,094	23,811	3,182	279
Age		(Years)	57 ± 9	60 ± 10	66 ± 8	69 ± 7
Body mass index		(kg/m^2^)	22.6 ± 2.9	23.3 ± 2.9	23.9 ± 3.0	23.7 ± 3.1
Systolic blood pressure		(mmHg)	135 ± 18	136 ± 17	140 ± 17	144 ± 19
Diastolic blood pressure		(mmHg)	80 ± 11	81 ± 11	82 ± 12	83 ± 12
Use of anti-hypertensive drugs		(%)	13.3	18.1	34.2	52.3
Use of hypoglycemic drugs		(%)	2.7	3.5	5.6	10.0
Total cholesterol		(mg/dL)	189 ± 35	193 ± 35	197 ± 35	193 ± 39
High-density lipoprotein		(mg/dL)	54.1 ± 15.5	54.1 ± 15.5	50.3 ± 15.5	46.4 ± 15.5
Use of lipid-lowering drugs		(%)	0.7	1.1	2.2	3.2
Current Smoking		(%)	43.6	36.5	24.5	20.1
Follow-up range		(Years)	0.2-25.7	0.1-25.7	0.1-25.7	0.3-25.7
Follow-up		(Person-years)	63,908	473,194	56,646	3,697
	Women					
Study size		(Persons)	3,831	43,809	10,982	562
Age		(Years)	54 ± 10	56 ± 10	66 ± 7	68 ± 8
Body mass index		(kg/m^2^)	23.2 ± 3.2	23.5 ± 3.2	24.0 ± 3.3	24.2 ± 3.6
Systolic blood pressure		(mmHg)	129 ± 18	131 ± 18	137 ± 17	141 ± 18
Diastolic blood pressure		(mmHg)	76 ± 10	78 ± 11	79 ± 10	80 ± 11
Use of anti-hypertensive drugs		(%)	10.8	15.7	35.4	51.8
Use of hypoglycemic drugs		(%)	1.6	1.8	3.6	5.7
Total cholesterol		(mg/dL)	201 ± 35	205 ± 35	213 ± 35	213 ± 43
High-density lipoprotein		(mg/dL)	58.0 ± 15.5	58.0 ± 15.5	54.1 ± 15.5	50.3 ± 15.5
Use of lipid-lowering drugs		(%)	2.1	2.8	5.2	4.3
Current smoker		(%)	2.0	1.8	1.1	1.1
Follow-up range		(Years)	0.1-25.7	0.1-25.7	0.1-25.7	0.4-25.7
Follow-up		(Person-years)	86,119	973,528	220,235	8,649

*eGFR* estimated glomerular filtration rate

The number of all-cause mortalities in this study population was 32,614 (15,039 and 17,575 for men and women, respectively), including 9,676 cancer mortalities (5,132 and 4,544 for men and women, respectively). Hazard ratios (HRs) and 95% confidence intervals (95% CIs) were calculated relative to the risk for individuals with eGFR 60-89 mL/min/1.73 m^2^ as reference. Incidents of cause-specific mortality due to cancer were defined by ICD codes 140-208 (C00-C97). As a secondary analysis, major site-specific cancers were investigated in which at least 50 cases were recorded. Survival time analysis was required because mean follow-up years widely ranged from 13.3 (CKD G3 or worse) to 20.7 (CKD G1) years in men and from 15.4 (CKD G3 or worse) to 22.4 (CKD G1) years in women (Table 1). Therefore, cancer-specific mortality was examined according to renal function at baseline by Cox proportional hazard modeling with adjustment for age and sex, and further with multivariable adjustment for blood pressure, anti-hypertensive treatment, current cigarette smoking, glucose tolerance status, diabetes treatment, alcohol intake, body mass index, serum total cholesterol, high-density lipoprotein-cholesterol, taking lipid-lowering drugs, and dipstick proteinuria (1+ or more). Table 2 presents numbers and adjusted HRs for any cancer mortality according to eGFR categories. Multivariable-adjusted HRs and 95%CIs for any cancer mortality in eGFR categories were 1.10 (95% CI, 1.02-1.18) for ≥90 mL/min/1.73 m^2^, 0.98 (95% CI, 0.93-1.03) for 45-59 mL/min/1.73 m^2^, and 1.14 (95% CI, 0.94-1.37) for <45 mL/min/1.73 m^2^, as compared with 60-89 mL/min/1.73 m^2^. Regarding site-specific cancers (Figure 1), the HRs for high-range renal function (eGFR ≥ 90 mL/min/1.73 m^2^) for bladder cancer were 2.25 (95% CI, 1.42-3.57) with adjustment for age and sex and 2.11 (1.32-3.37) with multivariable adjustment, while HRs for most cancer sites did not show high HRs, compared to eGFR 60-89 mL/min/1.73 m^2^.

**Table 2.** Numbers and Hazard Ratios of Cancer Mortalities among Different Grades of Renal Function.

eGFR, mL/min/1.73 m^2^	≥90	60-89	45-59	<45
Population	6,925	67,620	14,164	732
Mean follow-up length, years	21.7	21.4	19.5	14.7
Total mortalities	2,318	22,094	7,548	654
Cancer mortalities	779	6,986	1,799	112
Cancer death rate, per 10,000 person-years	51.9	48.3	65.0	90.7
Age- and sex-adjusted HR (95%CI)	1.14 (1.06-1.23)	Reference	0.98 (0.93-1.03)	1.15 (0.95-1.39)
Multivariable-adjusted HR (95%CI)	1.10 (1.02-1.18)	Reference	0.98 (0.93-1.03)	1.14 (0.94-1.37)

Multivariable adjustment was for blood pressure, anti-hypertensive treatment, cigarette smoking, status of glucose tolerance, diabetes treatment, alcohol intake, body mass index, serum total cholesterol, high-density lipoprotein-cholesterol, taking lipid-lowering drugs, and dipstick proteinuria (1+ or more). *HR* hazard ratio; *CI* confidence interval.

**Figure 1.**
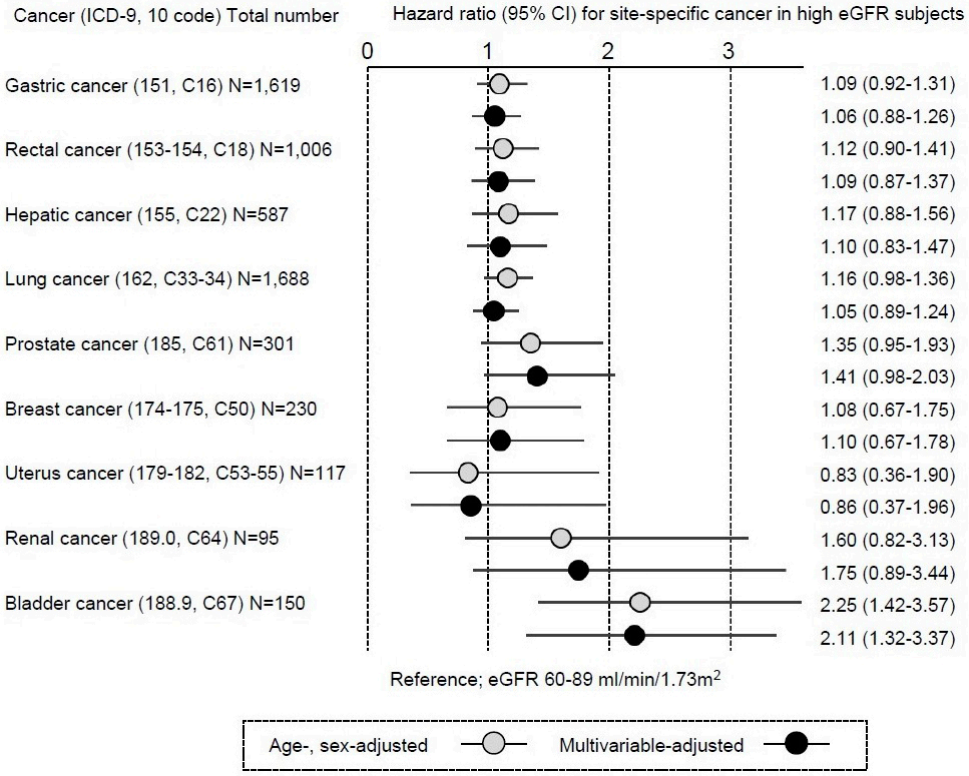
Hazard ratio for site-specific cancer mortality with high eGFR ≥ 90 mL/min/1.73 m^2^ Cancer-specific mortality was examined according to renal function at baseline by Cox proportional hazard modeling. Hazard ratios and 95% confidence intervals (CI) were calculated with adjustment for age and sex, and with multivariable adjustment for blood pressure, anti-hypertensive treatment, current cigarette smoking, status of glucose tolerance, diabetes treatment, alcohol intake, body mass index, serum total cholesterol, high-density lipoprotein-cholesterol, taking lipid-lowering drugs, and dipstick proteinuria (1+ or more).

The current study identified an excess risk of bladder cancer mortality among the high eGFR population. Previous studies consistently reported significantly higher HRs for cancer incidence among high eGFR groups (eGFR ≥ 90 mL/min/1.73 m^2^) and had postulated a J-shaped association between eGFR and cancer risk ^(4), (5), (6)^. In a Swedish cohort, the HR for any cancer with high eGFR (>104 mL/min/1.73 m^2^) was 1.09 (95% CI, 1.05-1.13) and that for urogenital cancer was 1.11 (95% CI, 0.93-1.32) ^(4)^. In a Korean cohort, HR for any cancer was lowest in subjects with eGFR 45-59 mL/min/1.73 m^2^ after multivariable adjustment, at 0.89 (95% CI, 0.78-1.00) as compared to high eGFR (eGFR ≥ 90 mL/min/1.73 m^2^) ^(5)^. In a cohort from the USA, HR for any cancer was associated with high eGFR (90-150 mL/min/1.73 m^2^) was 1.04 (95% CI, 1.01-1.06) ^(6)^. However, neither study reported bladder cancer-specific HRs among subjects with high eGFR, probably due to the sizes and designs of the studies ^(5), (6)^. After all, evidence for whether the findings on the risk of bladder cancer in this study are unique to Japan remains sparse.

In general, GFR increases during the disease course of diabetic nephropathy and obesity-related glomerulopathy because of an increase in filtration per glomeruli. This phenomenon is recognized as pathological glomerular hyperfiltration (GHF), rather than healthy eGFR elevation. Based on the background, most epidemiologic studies define a high eGFR as a proxy for GHF ^(4), (5), (6)^ If the high GFR in this study represents GHF and is implicated in the development of bladder cancer, important epidemiological evidence exists suggesting a possible association between GHF and bladder cancer in the Japanese population. Matsui et al. reported the HRs of any cancer mortality were 1.16 (95% CI, 1.03-1.31), 1.47 (95% CI, 1.27-1.70), and 1.61 (95% CI, 1.33-1.96) for trace, mild, and moderate-to-heavy dipstick proteinuria, respectively, in a community-based Japanese population ^(14)^. For urological cancer, markedly higher (~3, *p* < 0.001) HRs were shown in a population with mild proteinuria ^(14)^. In Cox proportional hazard modeling for the current study, multivariable adjustment included dipstick proteinuria (Table 2). However, the dipstick test is less sensitive to detecting trace amounts of urinary protein than quantitative methods ^(15)^. The results of the current study suggest that GHF not leading to positive dipstick proteinuria still carries an elevated risk of bladder cancer.

Regarding the relationship between high GFR and bladder cancer in this study, factors other than the adjustable risks (e.g., smoking, age, and blood pressure) may be considered as a possible explanation. Exposure to environmental and occupational carcinogens is a significant contributor to disease burden, and a recent systematic review attributed approximately 5%-6% of worldwide bladder cancer incidence to occupational carcinogen exposures ^(16)^. Specifically speaking, exposure to arsenic in drinking water and occupational exposure to aromatic amines and 4,4′-methylenebis (2-chloroaniline) are evident risk factors for bladder cancer ^(17)^. The type of such chemicals, the diseases, and the racial background of the exposed person may cause the urinary concentration through renal excretion or in the blood to behave in a J- or U-shape, not necessarily in a linear relationship with renal function ^(18), (19)^. Thus, situations may be observed in which the bladder is more susceptible to exposure to certain chemicals that may cause bladder cancer in a state of GHF. Further studies are required to better understand the pathophysiology involved because the actual mechanisms underlying the association between high eGFR and risk of bladder cancer mortality remains uncertain.

In showing an association between CKD and cancer mortality, a particular strength of this study was the use of a cohort from the general population with long-term follow-up. However, the current study also showed several limitations that must be considered. First, the present study did not account for the status of cancer diagnosis at baseline. Second, a possibility of detection bias exists in that bladder cancers were mostly detected early due to urinary abnormalities and consequent imaging tests (e.g., ultrasonography during the scrutiny of CKD for subjects with “mildly decreased GFR”; eGFR 60-89 mL/min/1.73 m^2^), facilitating early treatment. Third, a qualitative variable (i.e., presence or absence of current smoking) was used, but not the number of cigarettes smoked, for smoking habit in multivariable adjustment, and the amount of smoking and cancer mortality in the high eGFR population may not be accurately assessed.

In summary, a higher risk of bladder cancer mortality among individuals with eGFR of ≥90 mL/min/1.73 m^2^ was shown, suggesting that unhealthy GHF or nonadjustable risk factors in this study setting is partially involved in the prognosis of urological cancers.

## Article Information

### Conflicts of Interest

None

### Sources of Funding

This work was supported by the Ibaraki Prefectural Government and Grants-in-Aid from the Ministry of Health, Labour, and Welfare, Health and Labour Sciences Research Grants, Japan (Research on Health Services: H17-Kenkou-007; Comprehensive Research on Cardiovascular and Life-Style Related Diseases: H18-Junkankitou[Seishuu]-Ippan-012; Comprehensive Research on Cardiovascular and Life-Style Related Diseases: H20-Junkankitou[Seishuu]-Ippan-013; Intractable Diseases Conquest Research: H21-Nanchi-Ippan-059; Comprehensive Research on Cardiovascular and Life-Style Related Diseases: H23-Junkankitou[Seishuu]-Ippan-005; and Comprehensive Research on Cardiovascular and Life-Style Related Diseases: H26-Junkankitou [Seisaku]-Ippan-001; H29-Junkankitou-Ippan-003 and 20FA1002). This was also supported by a grant from the Japanese Association of Dialysis Physicians (grant no. #2020-3) and by the Japan Society for the Promotion of Science　grant nos. 18KK0431 and 19K17729.

### Author Contributions

Conceptualization, Investigation, and Writing - Original Draft Preparation: Kei Nagai

Supervision and Writing - Review & Editing: Kunihiro Yamagata, Toshimi Sairenchi, Kazumasa Yamagishi, Hiroyasu Iso, Fujiko Irie

### Approval by Institutional Review Board (IRB)

The Ibaraki Prefectural Health Study (IPHS) protocol was approved by the ethics committees of Ibaraki Prefecture (approval number R3-4) and the University of Tsukuba (#1628-1).

### Informed Consent

Informed consent to conduct an epidemiological study was obtained from community representatives. Individual consent was not required because the study analysis involved secondary use of data obtained for public health practice on cardiovascular disease prevention in the local community at that time. Adhering to relevant guidelines and regulations, participants were retrospectively allowed to withdraw their data from the analysis, and consent was considered to have been obtained if the participant did not decline to participate in this study.

### Data Availability

The datasets analyzed during the current study are not publicly available due to study protocol and strict privacy protection.
